# A sensitive green HPLC-fluorescence method for simultaneous analysis of sacubitril and valsartan in pure forms, pharmaceutical dosage form and human plasma

**DOI:** 10.1038/s41598-025-23181-x

**Published:** 2025-11-04

**Authors:** Lubna Ahmed Kormod, Maha A. Hegazy, Amira M. Kessiba

**Affiliations:** 1 Pharmaceutical Chemistry Department, Faculty of Pharmacy, Misr International University, KM 28, Cairo, Qalyubiyya Governorate Egypt; 2 Pharmaceutical Analytical Chemistry Department, Faculty of Pharmacy, Cairo University, Kasr-El Aini, Cairo, Egypt

**Keywords:** Sacubitril, Valsartan, HPLC, Fluorescence, Human plasma, Green, Chemistry, Medical research

## Abstract

The development of green methods of analysis has recently become a global trend in analytical chemistry, especially in routine HPLC analysis. This study presents a sensitive and economic green HPLC method with fluorescence detection for the simultaneous determination of sacubitril and valsartan in bulk, and pharmaceutical dosage forms in spiked human plasma using ibuprofen as an internal standard. Analysis was performed on a C18 column (150 mm × 4.6 mm, 5 mm) at ambient temperature with isocratic elution using 30 mM phosphate (pH 2.5) and ethanol in a ratio of (40:60 v/v) at a flow rate of 1.0 mL/min. The fluorescence detector was programmed for excitation and emission wavelengths from 0 to 3.2 min, where λ_Excitation_ = 250.0 nm and λ_Emission_ = 380.0 nm. Then the λ _Emission_ was changed to 320.0 nm from 3.2 to 5.2 min while the λ _Excitation_ was not changed. After 5.2 min, the λ _Excitation_ and λ _Emission_ were changed to 220.0 and 289.0 nm, respectively. Validation of the proposed method was performed in accordance with the ICH guidelines. The method showed good linearity for sacubitril and valsartan in the concentration ranges of 0.035 to 2.205 µg/mL and 0.035 to 4.430 µg/mL, respectively. The proposed method was proven eco-friendly and applicable for routine analysis of the studied drugs in human plasma through assessment by the Analytical Eco-Scale, AGREE, the complex GAPI, the AGSA, the CaFRI, the RGBfast and the Click Analytical Chemistry Index methods.

**Supplementary Information:**

The online version contains supplementary material available at 10.1038/s41598-025-23181-x.

## Introduction

Heart failure (HF) is a complicated, life-threatening cardiovascular condition; together with signs and symptoms resulting from impaired ventricular filling or ejection of blood, it has a high incidence of death and hospitalization that imposes a heavy burden on healthcare systems worldwide^1,2^.

The American College of Cardiology (ACC) and the American Heart Association (AHA) classified HF patients according to their left ventricular ejection fraction (LVEF) into HF with reduced ejection fraction (HFrEF, EF < 40%), HF with mild reduced ejection fraction (HFmrEF, EF = 40–50%), and HF with preserved ejection fraction (HFpEF, EF > 50%)^1^. However, all grades of symptomatic chronic HF show high rates of morbidities and mortalities^3^.

Recently, a new class of medications has been approved for patients with HFrEF, which is a combination of a 1:1 molar ratio of a neprilysin inhibitor (ARNIs, angiotensin II receptor-neprilysin inhibitors) and an angiotensin receptor blocker (ARB). In 2015, the Food and Drug Administration (FDA) and the European Medicines Agency (EMA) approved the complex of sacubitril (an ARNI) and valsartan (an ARB) that succeeded in delivering two pharmacologic effects^4,5^. Although the new combination sacubitril/valsartan was not used as a first-line treatment for HF because of its high costs, HF cases have been improved significantly, with fewer needs for indicating low therapeutic index digoxin or performing heart transplantation^6^.

**Fig. 1:**
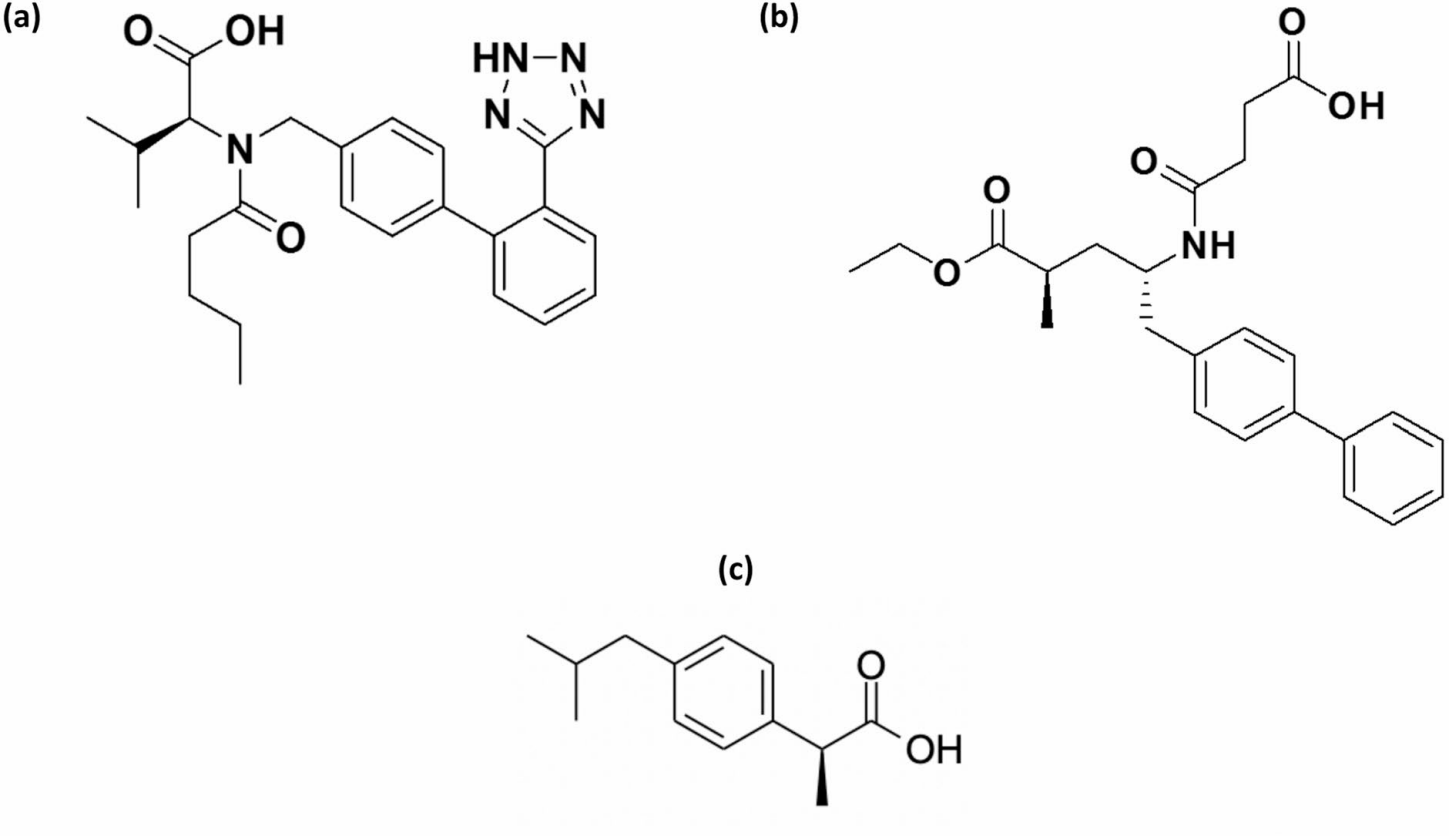
The chemical structures of (**a**) valsartan, (**b**) sacubitril, and (**c**) ibuprofen.

Chemically, valsartan (C_24_H_29_N_5_O_3_) is (2 S)-3-methyl-2- [pentanoyl -[[4-[2-(2 H-tetrazol-5-yl) phenyl] phenyl] methyl] amino] butanoic acid^7^ (Fig. 1a), while sacubitril (C_24_H_29_NO_5_) is 4-[[(2 S,4R)-5-ethoxy-4-methyl-5-oxo-1-(4-phenylphenyl)pentan-2-yl]amino]-4-oxobutanoicacid^8^ (Fig. 1b).

Due to the growing use of sacubitril/valsartan in HF treatment and its significant presence in the drug market, the development of validated analytical methods became potentially important.

Few spectrophotometric methods have been reported to analyze sacubitril/valsartan in a combined dosage form. A bivariate and two multivariate methods have been reported to determine the studied drugs in their pharmaceutical formulation^9^. Another method applied two manipulation approaches for the estimation of sacubitril/valsartan in their drug combination. The first approach was based on two wavelength selections in zero-order absorption spectra, and the second on ratio spectra^10^. Two spectrofluorimetric methods were also reported, where the first one depended on the calculation of the first derivative of the emission spectra^11^ and the second one was based on coupling synchronous fluorescence with derivative calculation of ratio spectra^12^.

Several chromatographic methods, including HPTLC, HPLC, and UPLC, were reported for analyzing sacubitril/valsartan in their dosage form, rat plasma, and spiked human plasma.

Two HPTLC methods were reported for analyzing the two drugs. The first method quantified sacubitril/valsartan in pharmaceutical formulations and spiked human plasma^13^, while the second determined the two drugs in raw materials and tablet formulations^14^.

Several stability-indicating HPLC methods were reported in the literature for the studied drugs. HPLC determined the two drugs in the presence of their stereoisomers and degradation products identified by LC-MS/MS^15^. Other stability-indicating HPLC methods were developed for sacubitril/valsartan in the presence of their impurities and degradation products^16,17^. Also, stability-indicating HPLC methods were reported to analyze the two drugs in the presence of their forced degradation products^18,19^. Other stability-indicating HPLC methods have also been reported to assay sacubitril/valsartan in pharmaceutical dosage form^20–25^. Additionally, a stereoselective normal phase HPLC method was reported to separate sacubitril/valsartan and their stereoisomeric impurities^26^.

The literature also introduced ultra-performance liquid chromatographic (UPLC) methods, such as simultaneous determination of sacubitril/valsartan in the presence of forced degradation products in bulk and pharmaceutical dosage form^27,28^ and in the presence of co-administered medications in human plasma^29^.

Several HPLC methods, including ion-pair HPLC^30^ and RP-HPLC^31–38^, were also reported for analyzing the two drugs in pharmaceutical dosage form.

HPLC with UV detection^39,40^, HPLC with fluorescence detection^41^, and LC-MS/MS^42^ have also been reported to detect sacubitril/valsartan in rat plasma, and a single HPLC-UV method has been reported for analyzing sacubitril/valsartan in human plasma^13^.

According to the reported HPLC methods, the proposed method has the advantage of providing a wider range of linearity (0.035–2.205 mg/mL and 0.035–4.430 mg/mL for sacubitril and valsartan, respectively), more cost-effectiveness due to the use of a traditional C18 column instead of special monolithic^41^ or cyano^40^ columns, and an isocratic pump instead of gradient elution^42^. Moreover, fluorescence detection increased the sensitivity of the proposed method compared to UV detection in the reported methods^13,39,40^. The reported LC-MS/MS^29,42^ methods also showed high sensitivity, but have higher costs, so the proposed method is simpler and more cost-effective.

Thus, the present work introduces an eco-friendly, simple, fast, sensitive, accurate, and economical HPLC method for simultaneously determining sacubitril and valsartan in pharmaceutical dosage form and human plasma.

## Experimental

### Instrumentation and software

“Agilent” HPLC instrument (1200 series) USA, equipped with an isocratic pump model G1310A, connected with a fluorescence detector model G1321A, was used for HPLC analysis. The injector was a manual Rheodyne injector (model G1328B, USA) equipped with a 20-µL injector loop and a 100-µL Agilent syringe. The instrument was connected to an IBM compatible PC bundled with Agilent Chemstation Chromatography data station software HPLC septum manager. A pH meter (Jenway 3510, UK), a sonicator (Soniclean 20 T, Australia), a membrane filter 0.45 μm (Alltech, USA), a vortex mixer (Stuart Scientific SA8, UK), and a cooling centrifuge (Sigma 3-30KS, Germany), were used in analysis.

### Chemicals and materials

All used solvents were of HPLC grade. Acetonitrile (Purity ≥ 99.9%), Ethyl alcohol (Purity ≥ 99.8%), Methyl alcohol (Purity ≥ 99.8%), and ultrapure HPLC grade water (Purity ≥ 18.2 MΩ·cm) were obtained from Sigma-Aldrich, Germany. All reagents were of analytical reagent grade, potassium dihydrogen phosphate (Purity ≥ 99.0%) and orthophosphoric acid (Purity ≥ 85.0%) were obtained from Adwic, Egypt. Standard sacubitril and valsartan (purities > 98.8%) were kindly supplied from Mash Premiere, Egypt. Standard Ibuprofen (purity > 99.1%) was supplied from Memphis, Egypt. The unidentifiable human plasma was purchased from VACSERA, Egypt, and Entresto^®^ tablets, manufactured by Novartis, Switzerland (Batch no. T0005), was purchased from the Egyptian market and was labeled to contain 49 mg sacubitril and 51 mg valsartan per tablet.

### Preparation of standard solutions and samples

#### Preparation of stock and working standard solutions

Stock standard solutions of 1.00 mg/mL of each of sacubitril (SAC), valsartan (VAL), and ibuprofen (IBU) were prepared by accurately transferring 0.1 g into 100-mL volumetric flasks, dissolving in a minimum amount of ethanol, and then completing to volume using ultra-pure deionized water. Working standard solutions were separately prepared by dilutions from the stock standard solutions using the mobile phase. All solutions were kept at 2–8^◦^C.

#### Preparation of pharmaceutical formulation solutions

Ten tablets of Entresto^®^ (Novartis, Switzerland, Batch no. T0005) were accurately weighed and finely powdered. Aliquots of the powdered tablets equivalent to 100 mg sacubitril/valsartan (SACVAL) complex were accurately weighed and suspended in 100.0 mL ethanol and sonicated for 30 min under controlled temperature. Solutions were then filtered, and the filtrate was then diluted with mobile phase to prepare a final concentration of 100 µg/mL of SACVAL complex.

#### Preparation of spiked human plasma samples

Aliquots of 960.0 µL of plasma were added to centrifugation tubes. Aliquots were spiked with 20.0 µL of each of SAC and VAL working standard or pharmaceutical formulation solutions, together with 20.0 µL of IBU the internal standard (IS) working standard solution of 100.00 µg/mL concentration. Then 4.0 mL of methanol were added to each. The tubes were vortex mixed for 1.0 min at 1350 rpm. The samples were then centrifuged at 5000 rpm for 15.0 min at 20 °C. The supernatant was diluted 1:1 using ultrapure water and a volume of 50.0 µL of the clear filtered supernatant was injected into the HPLC unit.

### General procedure

#### Chromatographic conditions

Chromatographic separation was performed on an Inertsil C_18_ column (150 mm × 4.6 mm, 5 μm; Japan). Column and injection temperatures were both maintained at ambient temperature. An isocratic system was operated at a 1.0 mL/min flow rate. The fluorescence detector was programmed for excitation and emission as presented in the following table.

**Table:** 

Time (min)	λ _Excitation_ (nm)	λ _Emission_ (nm)
0–3.20	250.0 nm	380.0 nm
3.2–5.2	250.0 nm	320.0 nm
˃ 5.2	220.0 nm	289.0 nm

#### Construction of calibration curves

Calibration standard solutions of spiked plasma were prepared from SAC and VAL working standard solutions at six different concentration levels, ranging from 0.035 to 2.205 µg/mL and 0.035 to 4.430 µg/mL, respectively. This incorporates the clinically relevant plasma concentration range^43^. Relative peak areas (RPAs) of both SAC and VAL to IBU were plotted against the corresponding concentrations. Least-squares linear regression analysis of the calibration data was performed using the linear equation y = ax + b, where y is the RPA, x is the analyte concentration, a is the slope, and b is the intercept.

## Results and discussion

### Method development and optimization

Optimization of chromatographic settings, including the pH selected for analysis, the organic modifier of the mobile phase, and the detection wavelengths, was achieved to elute peaks with high resolution and symmetry.

#### Selection of mobile phase

**Fig. 2:**
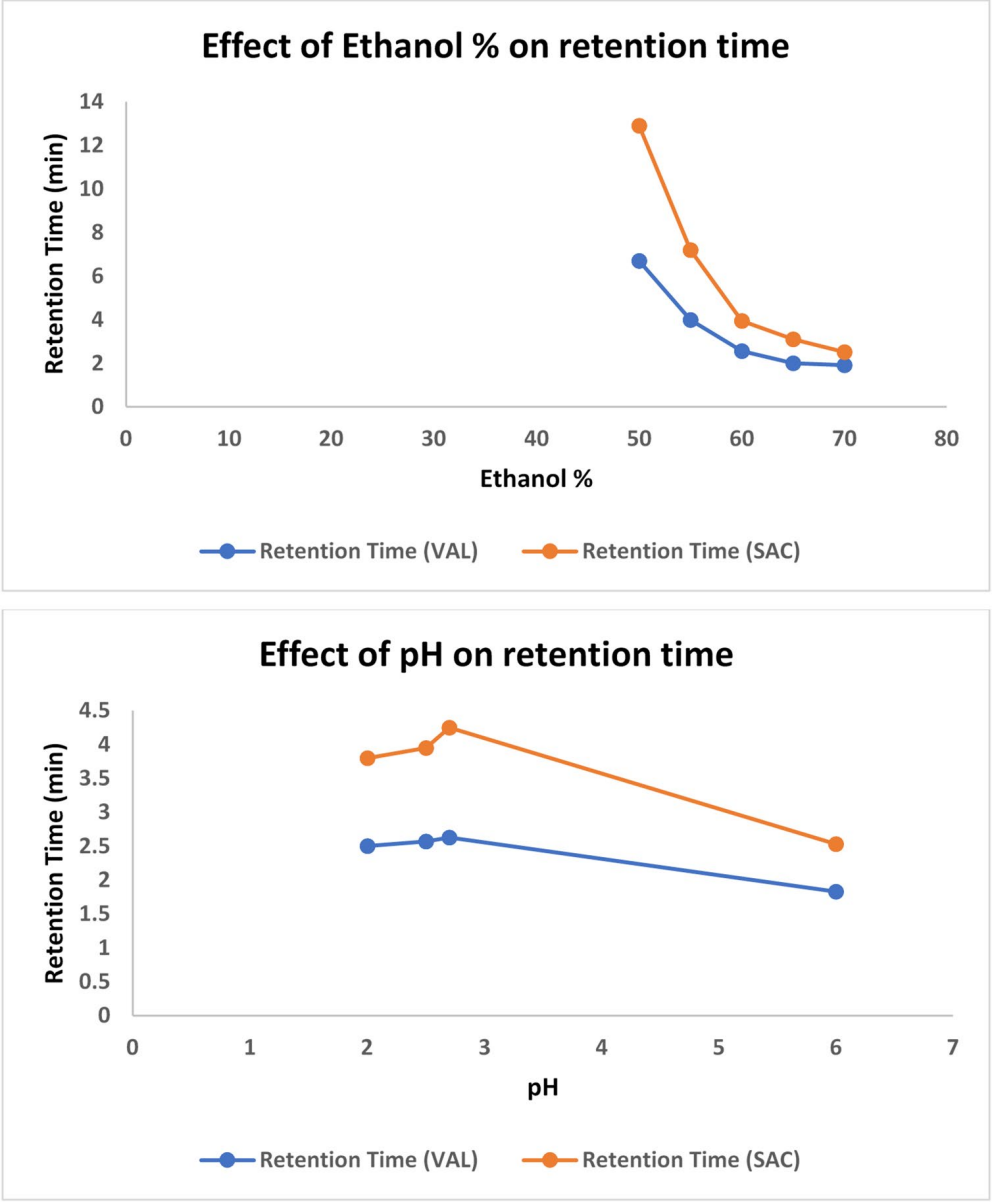
The effect of (**a**) ethanol%, and (**b**) pH in the mobile phase on retention times of SAC and VAL.

Different organic solvents were tried to attain optimal resolution within the shortest retention times for both drugs and the internal standard. Methanol was initially chosen as an organic modifier; however, it showed retarded retention times. Both acetonitrile and ethanol showed close and shorter retention times as they reduce polarity and weaken drug interactions with the stationary phase. However, Ethanol was preferentially chosen because of its more eco-friendly nature^44^. The phosphate buffer to ethanol ratio was tuned through various experiments to achieve optimal resolution of the three compounds. The ratio 40:60 (phosphate buffer: ethanol, v/v) was shown to optimize resolution, retention factor, peak symmetry, and analysis time. The effect of ethanol% in the mobile phase on the retention time of SAC and VAL is illustrated in Fig. 2a.

Different values of pH were tried, and the chosen pH was 2.5 for better symmetry and earlier elution, as well as being away from the pKa values of both SAC (pKa of 4.6) and VAL (pKa of 3.9 and 4.7)^45^ to avoid incomplete ionization. Since both drugs are weak acids, far above their pKa values, they become totally ionized, more hydrophilic and less interacting with the stationary phase. This results in elution at shorter retention times of both drugs, with a very early elution of VAL that overlaps with the solvent front. Going far below their pKa values, both drugs become fully unionized, more hydrophobic and more interacting with the stationary phase. This results in reasonable retention times of both, with acceptable retention factors and resolution. The effect of different pH values on the retention time through several trials is illustrated in Fig. 2b.

**Fig. 3:**
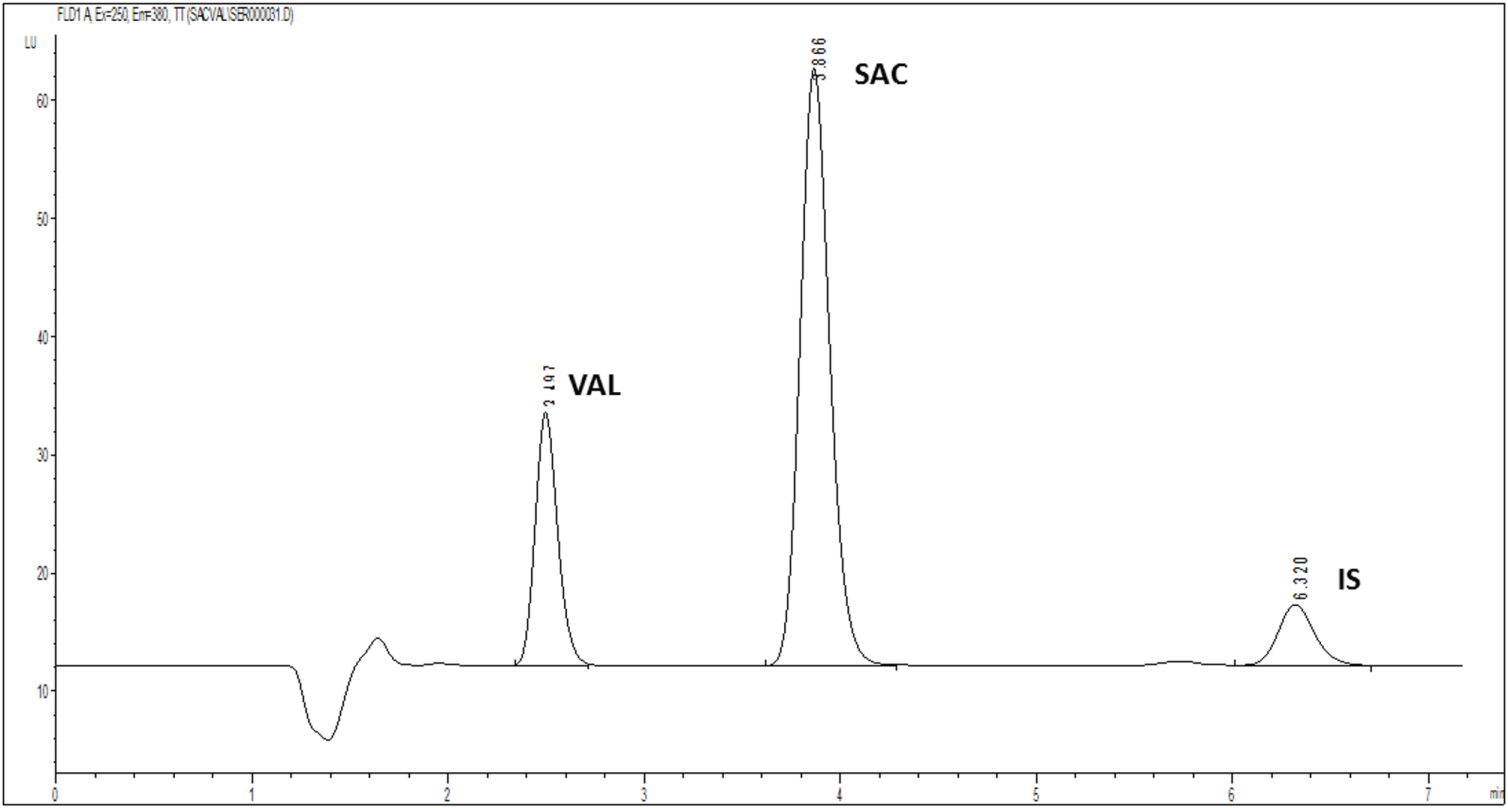
High performance liquid chromatograms of SAC, VAL and IS in their pure form using the proposed method.

Figure 3 shows the resulting chromatogram for the analysis of both SAC and VAL, as well as IBU as an IS, in their pure form using the proposed method.

#### Selection of the internal standard

Different compounds were tried as internal standards. However, the analysis using the chosen chromatographic conditions resulted in retention times overlapping with the analyzed drugs, even with reducing the percentage of the organic modifier, making resolution poor and quantification difficult. Ibuprofen was selected as the IS due to its relatively lower polarity, which allows higher retention on column and, in turn, longer retention time than SAC and VAL.

#### Plasma protein precipitation

Removing plasma proteins from plasma is a crucial step to avoid interference with the analyte of interest and damaging of the analytical column^46^. Protein precipitation was first tried using acetonitrile and methanol. As the polarity of an organic solvent increases, it becomes a less effective precipitating agent^47,48^. Methanol is more polar than acetonitrile and is therefore expected to be less effective at precipitating proteins. In related studies, protein precipitation with acetonitrile was the most used sample preparation method. However, trying both solvents resulted in better precipitation with acetonitrile in smaller volumes and poorer recoveries, but equivalent precipitation with methanol in larger volumes together with better recoveries. Protein precipitation was best achieved using a 1:4 ratio of plasma: methanol, by volume. Using less volume of methanol relative to plasma led to incomplete precipitation of plasma proteins. As chromatographic results are affected when large volumes of organic solvent are injected onto the HPLC column, causing tailing or extensive peak broadening^49^, dilution (1:1) with ultrapure water is carried out before injection and following vortexing and centrifugation steps.

### Method validation

To confirm the method’s suitability for its intended use, it was validated for specificity, linearity, precision, accuracy, limit of quantification, limit of detection, and robustness according to the international guidelines^50–52^.

#### Linearity

The proposed method’s linearity was determined by analyzing different concentration levels of each of SAC and VAL and determining their peak areas relative to that of the IS. The RPAs were correlated to the corresponding concentrations to construct the calibration curves. The linearity and parameters of the regression equation are listed in Table 1.

**Table 1:** Regression parameters of the proposed HPLC method for the simultaneous determination of SAC and VAL in spiked human plasma.

Parameter	SAC	VAL
Range (µg/mL)	0.035–2.205	0.035–4.430
Slope	59.262	18.823
Intercept	− 0.0725	0.0935
r^2^	0.9993	0.9995
LOD^(a)^ (µg/mL)	0.018	0.028
LOQ^(b)^ (µg/mL)	0.053	0.085

^a^LOD = (SD of the intercept /slope) × 3.3.

^b^LOQ = (SD of the intercept /slope) × 10.

#### Selectivity

**Fig. 4:**
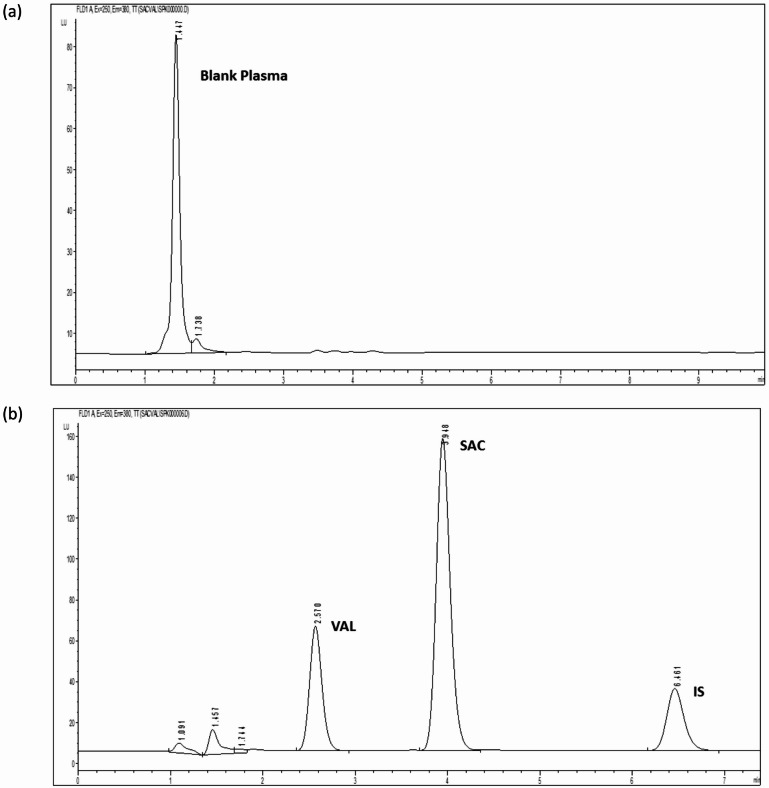
High performance liquid chromatograms of (**a**) blank human plasma, and (**b**) human plasma spiked with SAC, VAL and IS, using the proposed method.

The method’s specificity was determined by analyzing five human blank plasma samples, and it was confirmed by the absence of peaks at the retention times of SAC, VAL, and IS as represented in Fig. 4.

#### Precision

Intraday precision was evaluated by analyzing three concentrations of spiked plasma calibration standards in 3 replicates on the same day. The inter-day precision was determined by analyzing each calibration standard once for 3 consecutive days. Intra-day and inter-day precision were expressed as the percentage relative standard deviation (% RSD). The % RSD of SAC and VAL determined during the intraday and inter-day studies were all below 2.9%, as described in Table 2.

**Table 2:** Precision results of the proposed HPLC method for SAC and VAL in spiked human plasma.

Concentration (µg/mL)	Intra-day precision* (RSD%)		Inter-day precision* (RSD%)	
	SAC	VAL	SAC	VAL
0.07	0.92	1.57	2.33	2.88
0.13	0.72	1.31	1.99	2.87
0.88	0.56	1.04	2.23	2.81
Mean RSD%	0.73	1.31	2.18	2.85

*Average of 3 determinations.

#### Accuracy

Accuracy was expressed as the percentage recovery and was calculated as the measured value per the theoretical value and multiplied by 100. Analyte recovery was tested in triplicate for five concentrations (0.35, 0.44, 1.32, 1.77 and 2.22 µg/mL) for each of SAC and VAL. The quantitative recoveries of SAC and VAL in plasma ranged from 98.42% to 101.25% and 98.84% to 101.62%, respectively. The mean recoveries and standard deviations for all concentrations analyzed are given in Table 3.

**Table 3:** Accuracy results of the proposed HPLC method for SAC and VAL in spiked human plasma.

Concentration (µg/mL)	Recovery %* (Mean ± SD)	
	SAC	VAL
0.35	98.42 + 0.56	98.84 + 0.60
0.44	101.25 + 0.32	101.62 + 0.38
1.32	100.92 + 0.36	100.54 + 0.94
1.77	99.08 + 0.51	100.52 + 0.91
2.22	101.22 + 0.58	100.47 + 0.55
Mean ± SD	100.18 ± 1.33	100.40 ± 1.00

*Average of 3 determinations.

#### Limit of detection (LOD) and limit of quantification (LOQ)

The LOD values were 0.018 µg/mL and 0.028 µg/mL for SAC and VAL, respectively, while the LOQ values were 0.053 µg/mL and 0.085 µg/mL for SAC and VAL, respectively as illustrated in Table 1.

#### System suitability

System suitability tests are essential for HPLC methods^51^. These tests ensure that the chromatographic system adopted is suitable for the intended use. System suitability parameters including asymmetry factor, resolution, selectivity, retention factor, number of theoretical plates (N), and height equivalent theoretical plates (H)^51^ were investigated. Results obtained were satisfactory and are listed in Table 4.

**Table 4:** System suitability testing parameters of the proposed HPLC method for SAC and VAL in spiked human plasma.

Parameters	Obtained value			Reference value ^[51]^
	VAL	SAC	IBU	
Retention time, min	2.570	3.948	6.461	
Asymmetry factor	0.866	0.825	0.830	0.8–1.2
Retention factor, k	0.474	1.264	2.704	< 10
Resolution, R_s_	9.042	13.911		> 1.5
Selectivity, a	2.669	2.140		> 1
Number of theoretical plates, N	5175.136	9514.382	16798.450	Increase with efficiency of the separation
Height equivalent theoretical plates, H (cm/plate)	0.0029	0.0016	0.0009	The smaller the value the higher the column efficiency

#### Robustness

The proposed method’s robustness was tested. Several trials were conducted for the different flow rates, percentage of ethanol, and pH values. Retention times, the calculation of resolution, and the number of theoretical plates showed no significant change, as represented in Table 5, which proves the method’s consistency in routine laboratory work.

**Table 5:** Robustness results of the proposed HPLC method for SAC and VAL in their pure forms.

Parameter	VAL			SAC		
	t_R_*	Rs**	N***	t_R_*	Rs**	N***
Flow rate (− 0.1, 0.9 mL/min)	2.511	8.261	4940.250	3.77	14.182	8675.789
Flow rate (+ 0.1, 1.1 mL/min)	2.632	9.967	5427.843	4.151	14.531	10517.965
Ethanol % (+ 2%, 62%)	2.546	8.241	5078.931	3.802	13.756	8823.695
Ethanol % (− 2%, 58%)	2.643	8.957	5473.308	4.008	14.636	9805.770
pH 2.6 (+ 0.1)	2.544	8.963	5070.955	3.91	14.708	9332.109
pH 2.4 (− 0.1)	2.601	9.127	5300.737	3.992	13.501	9727.637

*Retention time, min.

**Resolution factor (Rs).

**Number of Theoretical Plates (N).

### Application to pharmaceutical Preparation

The proposed method was applied to determine the studied drugs in Entresto^®^ tablets, with mean percentage recoveries of 101.46 ± 0.18 and 102.84 ± 0.91 for SAC and VAL, respectively. The validity of the method was further assessed by applying the standard addition technique. The percentage recoveries, mean recovery and standard deviation are summarized in Table 6.

**Table 6:** Results of determination of SAC and VAL in Enteresto^®^ tablets and the application of standard addition technique in human plasma using the proposed HPLC method.

Pharmaceutical formulation	Component	Taken (µg/mL)	Found %* ± SD	Standard addition technique		
				Pure added (µg/mL)	Pure found (µg/mL)	Recovery%*
Enteresto^®^ tablets Batch no. T0005	SAC	0.96	101.46 ± 0.18	0.77	0.78	101.87
				0.96	0.97	100.65
				1.15	1.17	101.84
				Mean ± SD	101.45 ± 0.62	
	VAL	1.00	102.84 ± 0.91	0.80	0.81	101.13
				1.00	1.01	100.85
				1.20	1.22	101.27
				Mean *±* SD	101.08 *±* 0.15	

*Average of 3 determinations.

### Comparison with reported methods

Results obtained by the developed method were statistically compared to the reported one^13^ according to the ICH guidelines. The calculated t and F values were less than the tabulated ones, as shown in Table 7. This reveals a statistically non-significant difference between the two methods regarding accuracy and precision. The proposed method can then be used to quantitatively determine the studied drugs, in their pure forms and pharmaceutical dosage forms, in human plasma.

**Table 7:** Statistical comparison of the results obtained by applying the proposed HPLC method and the reported one^13^ in spiked human plasma, for determination of SAC and VAL in (a) their pure forms, and in (b) pharmaceutical dosage forms.

Parameter	SAC		VAL	
	Proposed method	Reported method*	Proposed method	Reported method*
(a)				
Mean	100.18	100.10	100.40	99.24
SD	1.33	0.91	1.00	0.48
Variance	1.77	0.83	1.00	0.23
n	5	5	5	5
Student’s t-test (2.306)**	0.103		1.642	
F value (5.32)**	2.12		4.34	
(b)				
Mean	101.46	101.08	102.84	100.59
SD	0.18	0.36	0.91	1.67
Variance	0.03	0.13	0.83	2.79
n	3	3	3	3
Student’s t-test (2.776)**	1.744		2.045	
F value (7.71)**	3.85		3.37	

*HPLC method carried out on C18 column (15 cm) using water with 0.1% triethylamine (pH 3.5 with phosphoric acid), methanol and ethanol (30:40:30, v/v) as a mobile phase and a UV detector at wavelength of 267 nm.

**Figures in parenthesis are the corresponding tabulated values at *P* = 0.05.

### Evaluation of greenness of the developed HPLC method

One of the most important parameters to consider when developing a new analytical method is its greenness^53^. The optimum greenness is achieved using minimum reagents and energy and disposing fewer wastes. The developed method greenness assessment was calculated using the Analytical Eco-scale^54^, AGREE calculator^55^, Complex MoGAPI^56^, CaFRI^57^, and AGSA^58^ methods.

#### Analytical eco-scale

Based on the Analytical Eco-scale^54^, ideal methods have scores equal to 100, excellent when higher than 75, acceptable when more than 50, and inadequate if less than 50. The Analytical Eco-scale is calculated as shown in the following equation,

$${\text{Analytical Eco-Scale}}=100 - {\text{sum of penalty points}}$$

Calculating the total penalty points depends on some parameters, including the types of reagents used, their proportions, types of instruments, occupational hazards, and the type and amount of waste.

The Eco-scale score of the developed method was 66, as illustrated in Table 8, therefore the developed method is acceptable regarding the greenness approach.

**Table 8:** Eco-Scale penalty points (PPs) for the determination of SAC and VAL using the proposed HPLC method.

Parameters	Penalty points			
Reagents	Amount PPs	Hazard PPs (Pictograms x Signal word)		Total PPs
		Pictograms	Signal word (warning/danger)	
Ethanol	3	2	2	12
Methanol	2	3	2	12
Water	3	0	0	0
Potassium dihydrogen phosphate	1	0	1	0
Phosphoric acid	1	1	2	2
Instrument (HPLC)	Methods using 0.1–1.5 kWh per sample			1
Occupational hazard	Analytical process hermitization			0
Waste	Amount > 10 mL			5
	No treatment			3
Total penalty points				S 34
Analytical Eco-Scale total score * = 100 − sum of penalty points				66

*Analytical Eco-Scale score, ideal method if = 100, excellent > 75, acceptable > 50, inadequate < 50.

#### AGREE calculator

**Fig. 5:**
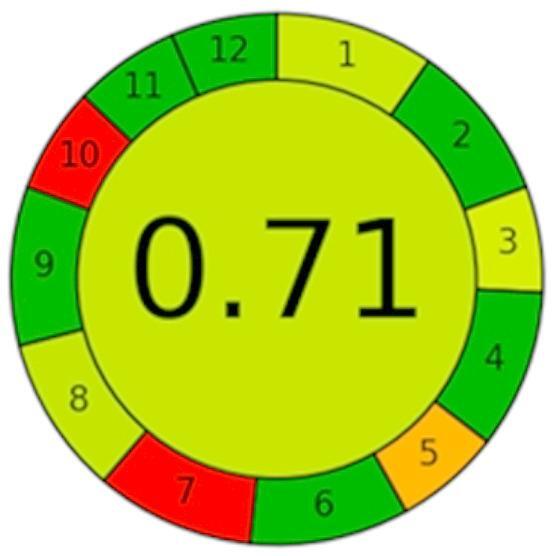
The AGREE calculator results for the proposed HPLC method.

The AGREE calculator^55^ is a simple, free tool for assessing the greenness of analytical methods. The assessment relies on 12 principles of greenness and uses a scale from 0 to 1. Results are presented in pictograms with a circle in the middle to show the final score. The green color intensity increases as the score gets closer to 1. The pictogram scores of the AGREE calculator for the developed method in plasma is 0.71. Thus, the proposed method offers an acceptable greenness as shown in Fig. 5.

#### Complex MoGAPI

**Fig. 6:**
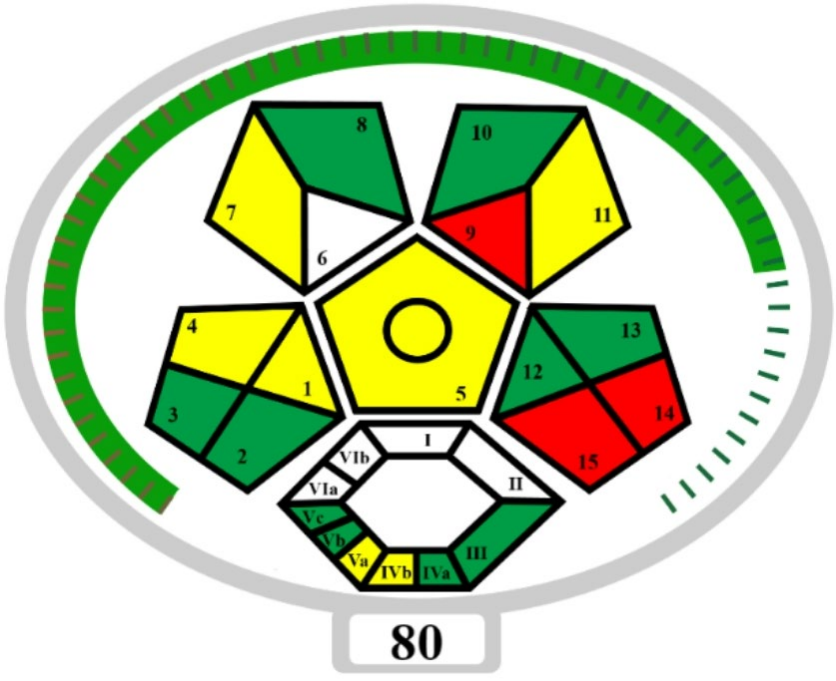
The complex MoGAPI score for the proposed HPLC method.

The Complex Modified GAPI (Complex MoGAPI)^56^ is derived from the former introduced tools: Green Analytical Procedure Index (GAPI)^59^, Complex GAPI^60^, and Modified GAPI^61^ methods. The GAPI^59^ method is designed to evaluate the environmental effect of analytical methods through five colored pentagrams. Each pentagram represents different stages of the analytical method, sampling, method type, sample preparation, solvent, and reagent use, as well as the consumption of energy. The complex GAPI^60^ method introduced an additional histogram to the GAPI plot for further qualitative assessments. The main drawback of these tools is the absence of a total score, making it difficult to compare methods. The Modified GAPI^61^ tool then combines the visual representation of GAPI^59^ with the calculation of the total score of the analytical eco-scale^54^ method. The complex MoGAPI combines the modification of MoGAPI^61^ with the complex GAPI^60^ representations, resulting in enhanced quantitative comparisons. Each step’s credit is based on its environmental impact; the greener the method, the higher its score is. To ensure fair results, non-applicable steps are excluded, then the total score is converted into a percentage, which is used to categorize method greenness. The software was used to evaluate the greenness of the developed method. It produced a visual pentagram with a greenness score of 80, reflecting a method with high greenness shown in Fig. 6.

#### Carbon footprint reduction index (CaFRI)

**Fig. 7:**
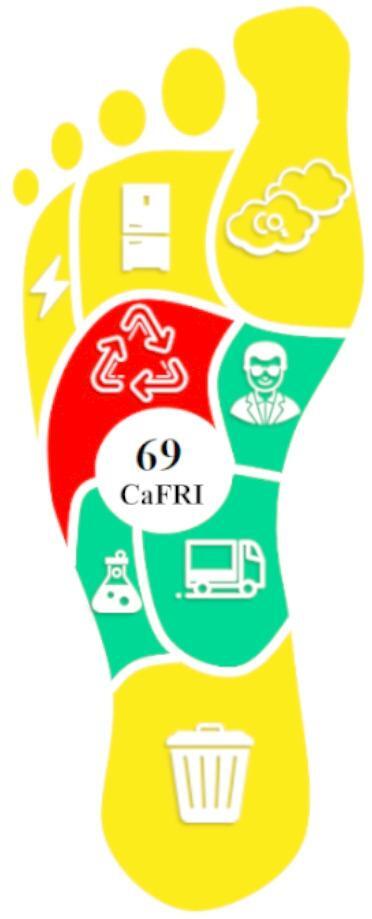
The CaFRI score for the proposed HPLC method.

The Carbon Footprint Reduction Index (CaFRI)^57^, a recently developed online tool, was introduced to evaluate, and improve the sustainability of analytical techniques, considering the carbon footprint’s primary environmental impact. The tool considers factors such as energy control measures, carbon footprint reduction measures, storage, transportation, personnel, waste disposal and recycling, and use of chemicals. The software questionnaire evaluates each factor and assigns corresponding points concerning the carbon footprint. Points are then converted to a final score on a scale of 0-100. The tool results are then presented in a human foot-shaped pictogram. Like the concept of green chemistry, different areas on the pictogram represent the corresponding carbon footprint criteria. Red, yellow and green colors correspond to poor, average, and green ratings, respectively. The software was used to evaluate the greenness of the proposed method and produced a pictogram with a greenness score of 69, which reflects a method with high greenness, as shown in Fig. 7.

#### Analytical green star area (AGSA)

**Fig. 8:**
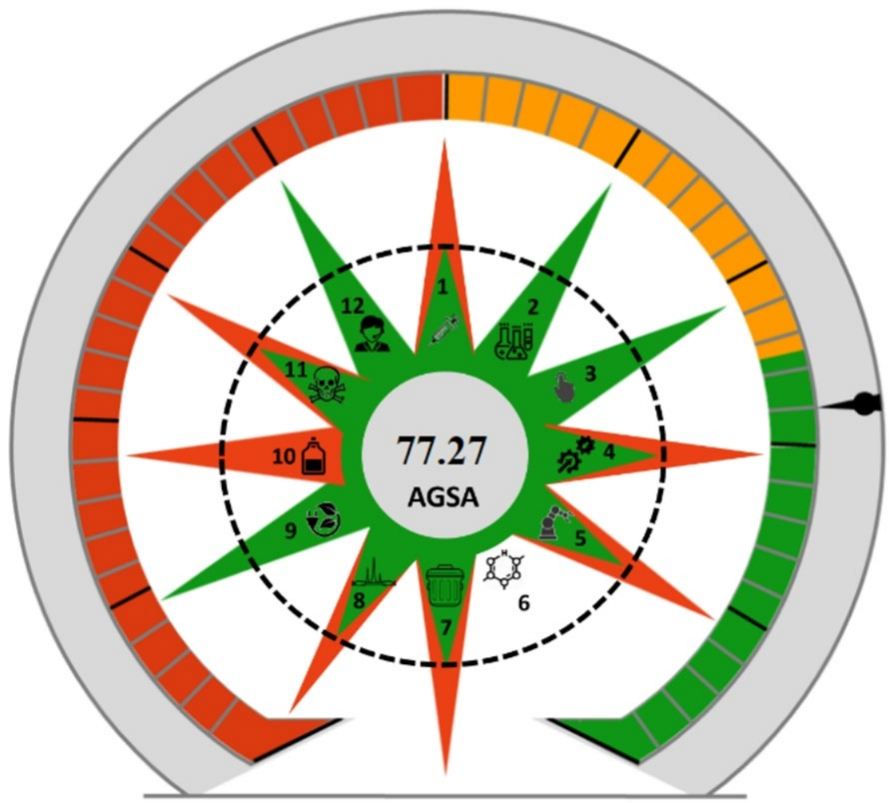
The AGSA score for the proposed HPLC method.

The AGSA metric evaluates analytical methods’ greenness by scoring their alignment with the 12 green analytical chemistry principles^58^. A simple software presents specific multiple-choice questions per principle, allowing for gradual discrimination between methods. Greater sustainability is indicated through higher scores, obtained through minimal sample treatment, lower consumption of energy, the use of non-hazardous reagents, and waste management strategies^58^. These calculated points are cumulative, summed up to a 36 total points (3 points for 12 principles), and the results are expressed as percentages. The software was used to evaluate the greenness of the proposed method and produced a plot with a greenness score of 77.27%, which reflects a method with high greenness, as shown in Fig. 8.

### Evaluation of whiteness of the developed HPLC method using RGBfast method

**Fig. 9:**
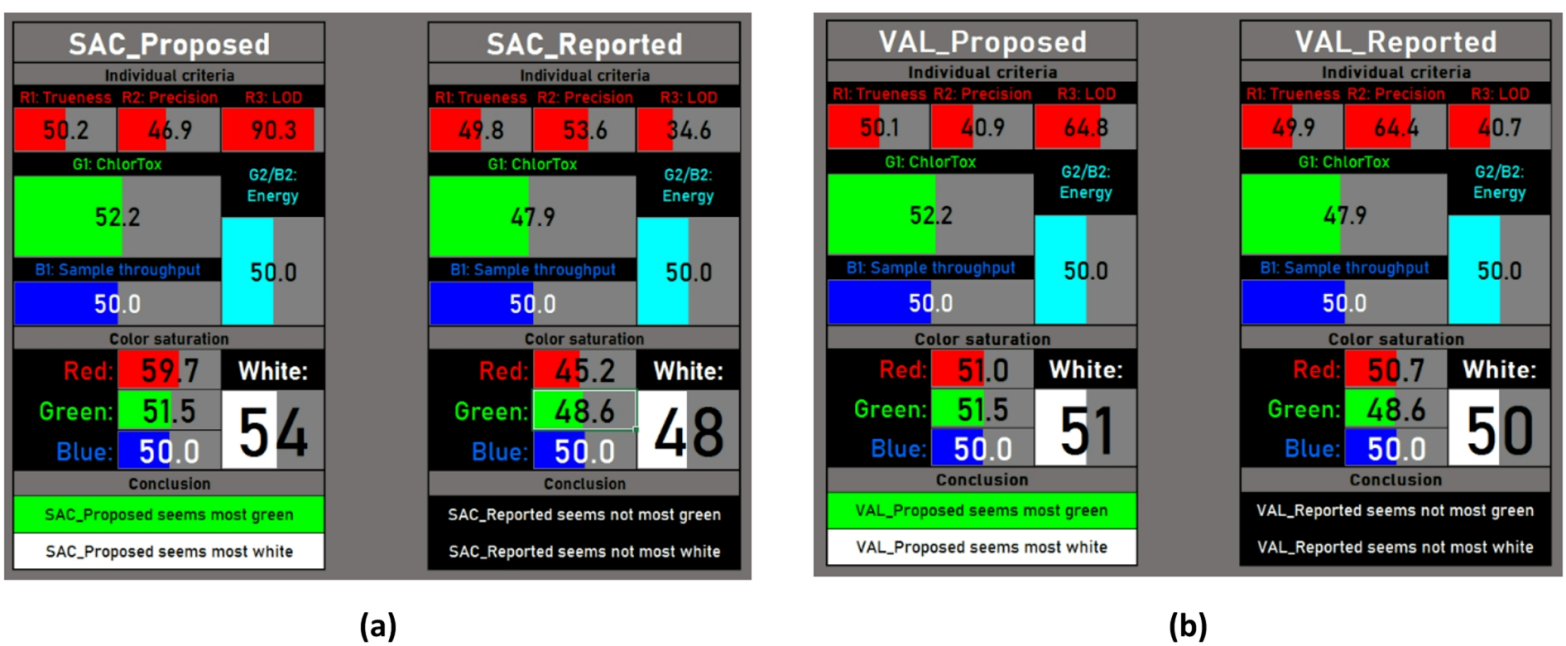
The automatically generated RGBfast tables to compare the proposed and reported methods for (**a**) SAC and (**b**) VAL.

The RGBfast method^62^ evaluates the proposed method’s greenness and whiteness. It is a simplified tool that rates analytical methods by assessing their environmental impact through color codes. Red codes for less green, green for eco-friendly, and blue for balanced method profiles. This simple tool can help assess and improve the sustainability of methods. The RGBfast model develops an automated Excel spreadsheet in which analysts fill in the method-relevant data. This simple model evaluates analytical methods through six criteria that balance effectiveness, sustainability, and efficiency, making evaluations quicker and easier. The model was applied to both the proposed and the reported^13^ methods, as shown in Fig. 9. The developed method showed higher green and white profiles than the reported one.

### Practicality and applicability of the proposed method

**Fig. 10:**
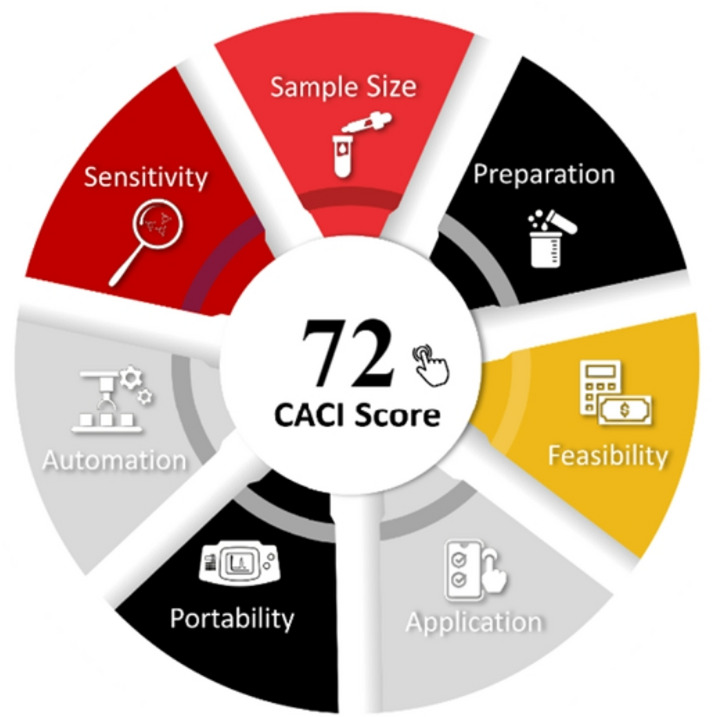
The CACI score of the proposed HPLC method.

This study introduces the Click Analytical Chemistry Index (CACI)^63^, a free software for evaluating validated analytical methods, regarding their practicality and usability. The performance of the evaluated method is presented through a pictogram with color codes: Excellent performance is denoted through colored areas. In contrast, grey indicates moderate performance, and black represents non-compliance. This tool allows analysts to identify the strengths and weaknesses of methods. The CACI score of the proposed HPLC-FD method was found to be 72, which is acceptable in terms of practicality, as shown in Fig. 10.

## Conclusion

An isocratic RP-HPLC method coupled to fluorescence detection was developed to quantify sacubitril and valsartan in human plasma effectively. The proposed method provides high sensitivity over wide concentration ranges and good accuracy and precision. Distinct calibration curves were constructed for both SAC and VAL. The ICH requirements were followed for the analytical method validation of the procedure. The constructed calibrations were applied for the determination of the pure analytes as well as their dosage forms in human plasma. The proposed approach was eco-friendly, simple, rapid, and sensitive; therefore, it is recommended for measurement of the investigated analytes in human plasma. The obtained results make the proposed method a promising tool for therapeutic drug monitoring, bioequivalence, and pharmacokinetic studies.

## Supplementary Information

Below is the link to the electronic supplementary material.

Supplementary Material 1

## Data Availability

The data that has been analyzed and used in this study has been presented in this research and any other data will be available from the corresponding author upon reasonable request.
