# Supplementary figures and images for: A sensitive green HPLC-fluorescence method for simultaneous analysis of sacubitril and valsartan in pure forms, pharmaceutical dosage form and human plasma
Source: Sci Rep. 2025 Nov 4;15:38484. doi: 10.1038/s41598-025-23181-x (PMC12586510; doi:10.1038/s41598-025-23181-x)

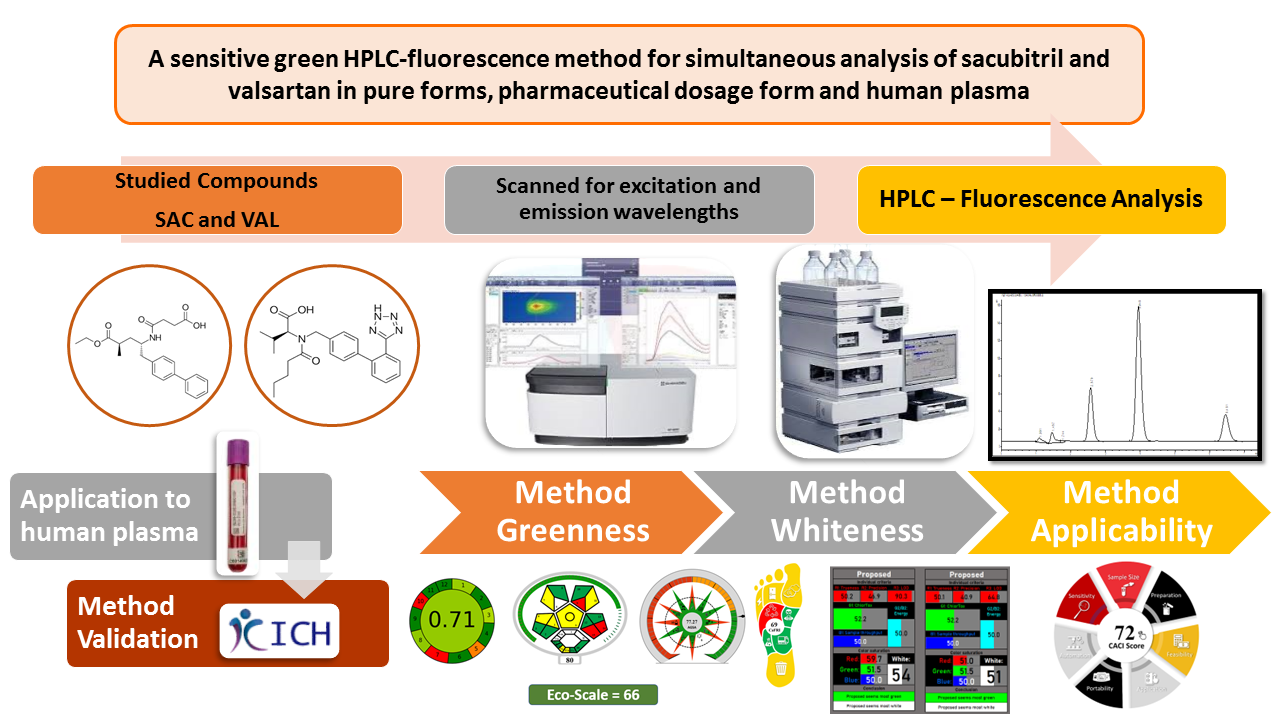

Supplement: Supplementary file 1 — Supplementary Material 1 [file 41598_2025_23181_MOESM1_ESM.tif]
